# Poly[μ-chlorido-[μ_4_-5-(4-pyrid­yl)tetra­zol­ato]dicopper(I)]

**DOI:** 10.1107/S1600536809006564

**Published:** 2009-03-06

**Authors:** Cun-Kuan Wang, Xiao-Yan Li

**Affiliations:** a Yangming School, Ningbo University, Ningbo, Zhejiang 315211, People’s Republic of China; b Department of Biological Engineering, Zibo Vocational Institute, Zibo, Shandong 255314, People’s Republic of China

## Abstract

The title three-dimensional coordination polymer, [Cu_2_Cl(C_6_H_4_N_5_)]_*n*_, is the product of the hydro­thermal reaction of CuCl_2_·2H_2_O and 5-(4-pyrid­yl)-1*H*-tetra­zole (4-Hptz). The two independent Cu^I^ ions are coordinated in distorted tetra­hedral and distorted trigonal coordination environments. In the unique 5-(4-pyrid­yl)-1*H*-tetra­zolate ligand, the dihedral angle between the pyridine and tetra­zole rings is 17.3 (2)°.

## Related literature

For related transition metals complexes of 5-(4-pyrid­yl)-1*H*-tetra­zole, see: Xue *et al.* (2002 ▶); Jiang *et al.* (2004 ▶); Luo *et al.* (2005 ▶); Lin *et al.* (2005 ▶); Chen *et al.* (2008 ▶). For the applications of tetra­zoles, see: Butler (1996 ▶).
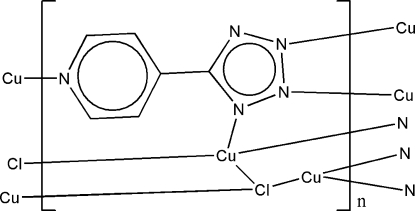


## Experimental

### 

#### Crystal data

[Cu_2_Cl(C_6_H_4_N_5_)]
*M* *_r_* = 308.67
Monoclinic, 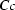

*a* = 19.6899 (7) Å
*b* = 3.64790 (10) Å
*c* = 11.6337 (3) Å
β = 102.923 (2)°
*V* = 814.45 (4) Å^3^
*Z* = 4
Mo *K*α radiation
μ = 5.50 mm^−1^
*T* = 298 K
0.30 × 0.26 × 0.24 mm

#### Data collection

Bruker SMART APEXII diffractometer
Absorption correction: multi-scan (*SADABS*; Sheldrick, 1996 ▶) *T*_min_ = 0.230, *T*_max_ = 0.269
3752 measured reflections
1572 independent reflections
1415 reflections with *I* > 2σ(*I*)
*R*_int_ = 0.027

#### Refinement

*R*[*F* ^2^ > 2σ(*F* ^2^)] = 0.027
*wR*(*F* ^2^) = 0.092
*S* = 1.11
1572 reflections
128 parameters
2 restraints
H-atom parameters constrained
Δρ_max_ = 0.71 e Å^−3^
Δρ_min_ = −0.71 e Å^−3^
Absolute structure: Flack (1983 ▶), 621 Friedel pairs
Flack parameter: 0.19 (3)

### 

Data collection: *APEX2* (Bruker, 2003 ▶) ; cell refinement: *SAINT* (Bruker, 2003 ▶); data reduction: *SAINT*; program(s) used to solve structure: *SHELXS97* (Sheldrick, 2008 ▶); program(s) used to refine structure: *SHELXL97* (Sheldrick, 2008 ▶); molecular graphics: *SHELXTL* (Sheldrick, 2008 ▶); software used to prepare material for publication: *SHELXL97*.

## Supplementary Material

Crystal structure: contains datablocks global, I. DOI: 10.1107/S1600536809006564/lh2752sup1.cif

Structure factors: contains datablocks I. DOI: 10.1107/S1600536809006564/lh2752Isup2.hkl

Additional supplementary materials:  crystallographic information; 3D view; checkCIF report

## Figures and Tables

**Table:** 

Cu1—N3^i^	1.958 (5)
Cu1—N1	2.038 (5)
Cu1—Cl1	2.4422 (15)
Cu1—Cl1^ii^	2.5090 (16)
Cu2—N2^iii^	1.921 (5)
Cu2—N5^iv^	1.931 (4)
Cu2—Cl1	2.4923 (18)

**Table:** 

N3^i^—Cu1—N1	133.4 (2)
N3^i^—Cu1—Cl1	116.27 (15)
N1—Cu1—Cl1	97.70 (14)
N3^i^—Cu1—Cl1^ii^	106.89 (15)
N1—Cu1—Cl1^ii^	100.51 (13)
Cl1—Cu1—Cl1^ii^	94.90 (6)
N2^iii^—Cu2—N5^iv^	152.3 (2)
N2^iii^—Cu2—Cl1	101.13 (17)
N5^iv^—Cu2—Cl1	106.30 (16)
Cu1—Cl1—Cu2	123.48 (7)
Cu1—Cl1—Cu1^iii^	94.90 (6)
Cu2—Cl1—Cu1^iii^	78.17 (5)

Symmetry codes: (i) 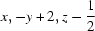; (ii) 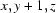; (iii) 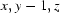; (iv) 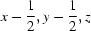.

**Fig. 1.**
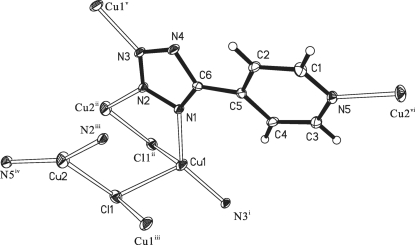
View of the coordination environment around the CuI ions and 4-ptz ligand of title complex with labeling scheme and 30% thermal ellipsoids. Symmetry codes: (i) x, -y + 2, z - 1/2; (ii) x, y + 1, z; (iii) x, y - 1, z; (iv) x - 1/2, y - 1/2, z;(v) x, -y + 2, z + 1/2; (vi) x + 1/2, y + 1/2, z.

**Fig. 2.**
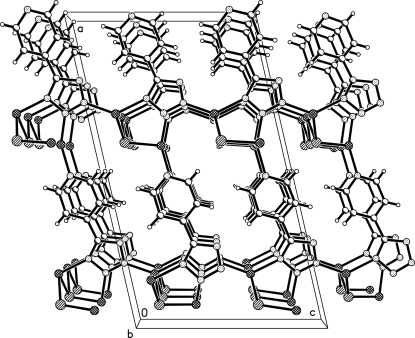
Part of the crystal structure of the title complex.

## supplementary crystallographic information

### Comment

Tetrazoles have found a wide range of applications in areas as diverse as coordination chemistry, medicinal chemistry and materials science (Butler, 1996). The study of complexes containing substituted tetrazole ligands is of interest to delineate the ways in which tetrazoles bind to metal centres. Recently, a series of 5-(4-pyridyl)-1*H*-tetrazole complexes of transition metals have been reported in which a range of coordination modes for the ligand were observed and extended two-dimensional and three-dimensional structures identified (Xue *et al.*, 2002; Jiang *et al.*, 2004; Luo *et al.*, 2005; Lin *et al.*, 2005; Chen *et al.*, 2008). Herein, we report the crystal structure of a three-dimensional coordination polymer, [Cu^I^_2_Cl(4-ptz)]_n_, derived from 5-(4-pyridyl)-1*H*-tetrazole and CuCl_2_.2H_2_O under hydrothermal reaction.

The asymmetric unit of the title complex contains of two independent Cu^I^ ions, one Cl^-^, and one 4-ptz ligand. As shown in Fig. 1, atom Cu1 adopts distorted tetrahedral geometry with a Cl_2_N_2_ donor set and atom Cu2 is in a disorted trigonal coordination geometry with an N_2_Cl donor set. Atom Cl1 is bonded to three Cu^I^ atoms, and the 4-ptz ligand coordinates to four Cu^I^ ions. It is noteworthy that atoms N1, N2, and N3 bond to three Cu^I^ atoms, respectively, forming a *µ*_3_-1,2,3-tetrazolyl coordination mode. The overall structure of title complex is a three-dimensional network (Fig. 2).

### Experimental

A mixture of CuCl_2_.2H_2_O (0.172 g, 1 mmol), 5-(4-pyridyl)-1*H*-tetrazole (0.074 g, 0.5 mmol) in 8 ml deionized water was homogenized at room temperature for 30 minutes. Then the final solution was sealed in a 20 mL stainless-steelautoclave at 433 K for 72 h. A quantity of crystals was obtained after the solution was cooled to room temperature. The crystals were filtered, washed with deionized water and dried at room temperature. The yield is *ca* 64% based on CuCl_2_.2H_2_O.

### Refinement

All H atoms on C atoms were positioned geometrically and allowed to ride on their respective parent atoms, with C—H = 0.93 and *U*_iso_(H) = 1.2 *U*_eq_(C). The crystal is an inversion twin with the ratio of twin components 0.81 (3):0.19 (3).

### Crystal data

**Table:**

[Cu_2_Cl(C_6_H_4_N_5_)]	*F*(000) = 600
*M**_r_* = 308.67	*D*_x_ = 2.517 Mg m^−^^3^
Monoclinic, *C**c*	Mo *K*α radiation, λ = 0.71073 Å
Hall symbol: C -2yc	Cell parameters from 1367 reflections
*a* = 19.6899 (7) Å	θ = 2.1–27.8°
*b* = 3.6479 (1) Å	µ = 5.50 mm^−^^1^
*c* = 11.6337 (3) Å	*T* = 298 K
β = 102.923 (2)°	Block, yellow
*V* = 814.45 (4) Å^3^	0.30 × 0.26 × 0.24 mm
*Z* = 4	

### Data collection

**Table:**

Bruker SMART CCD APEXII diffractometer	1572 independent reflections
Radiation source: fine-focus sealed tube	1415 reflections with *I* > 2σ(*I*)
graphite	*R*_int_ = 0.027
Detector resolution: 8.40 pixels mm^-1^	θ_max_ = 27.8°, θ_min_ = 2.1°
ω scans	*h* = −21→25
Absorption correction: multi-scan (*SADABS*; Sheldrick, 1996)	*k* = −4→4
*T*_min_ = 0.230, *T*_max_ = 0.269	*l* = −15→15
3752 measured reflections	

### Refinement

**Table:**

Refinement on *F*^2^	Secondary atom site location: difference Fourier map
Least-squares matrix: full	Hydrogen site location: inferred from neighbouring sites
*R*[*F*^2^ > 2σ(*F*^2^)] = 0.027	H-atom parameters constrained
*wR*(*F*^2^) = 0.092	*w* = 1/[σ^2^(*F*_o_^2^) + (0.0547*P*)^2^] where *P* = (*F*_o_^2^ + 2*F*_c_^2^)/3
*S* = 1.11	(Δ/σ)_max_ < 0.001
1572 reflections	Δρ_max_ = 0.71 e Å^−^^3^
128 parameters	Δρ_min_ = −0.71 e Å^−^^3^
2 restraints	Absolute structure: Flack (1983), 621 Friedel pairs
Primary atom site location: structure-invariant direct methods	Flack parameter: 0.19 (3)

### Special details

**Table:**

Geometry. All e.s.d.'s (except the e.s.d. in the dihedral angle between two l.s. planes) are estimated using the full covariance matrix. The cell e.s.d.'s are taken into account individually in the estimation of e.s.d.'s in distances, angles and torsion angles; correlations between e.s.d.'s in cell parameters are only used when they are defined by crystal symmetry. An approximate (isotropic) treatment of cell e.s.d.'s is used for estimating e.s.d.'s involving l.s. planes.
Refinement. Refinement of *F*^2^ against ALL reflections. The weighted *R*-factor *wR* and goodness of fit *S* are based on *F*^2^, conventional *R*-factors *R* are based on *F*, with *F* set to zero for negative *F*^2^. The threshold expression of *F*^2^ > σ(*F*^2^) is used only for calculating *R*-factors(gt) *etc*. and is not relevant to the choice of reflections for refinement. *R*-factors based on *F*^2^ are statistically about twice as large as those based on *F*, and *R*- factors based on ALL data will be even larger.

### Fractional atomic coordinates and isotropic or equivalent isotropic displacement parameters (Å^2^)

**Table:**

	*x*	*y*	*z*	*U*_iso_*/*U*_eq_	
Cu1	0.17283 (4)	0.9867 (2)	0.16168 (5)	0.0302 (2)	
Cu2	0.07924 (5)	0.1504 (3)	0.34304 (8)	0.0341 (2)	
Cl1	0.08796 (8)	0.4991 (4)	0.16267 (13)	0.0264 (3)	
N1	0.2207 (2)	0.9667 (13)	0.3361 (4)	0.0188 (9)	
N2	0.1753 (3)	1.0294 (14)	0.4064 (5)	0.0209 (9)	
N3	0.2072 (3)	0.9615 (14)	0.5171 (4)	0.0219 (10)	
N4	0.2723 (3)	0.8552 (15)	0.5223 (4)	0.0232 (10)	
N5	0.4827 (2)	0.6742 (14)	0.3528 (4)	0.0215 (10)	
C1	0.4640 (3)	0.5674 (16)	0.4514 (6)	0.0234 (12)	
H1A	0.4971	0.4535	0.5101	0.028*	
C2	0.3980 (3)	0.6184 (16)	0.4700 (5)	0.0203 (11)	
H2A	0.3876	0.5450	0.5407	0.024*	
C3	0.4326 (3)	0.8253 (16)	0.2677 (5)	0.0230 (11)	
H3A	0.4446	0.9003	0.1985	0.028*	
C4	0.3644 (3)	0.8755 (16)	0.2771 (5)	0.0203 (11)	
H4A	0.3312	0.9704	0.2145	0.024*	
C5	0.3469 (3)	0.7798 (14)	0.3829 (5)	0.0176 (10)	
C6	0.2791 (3)	0.8611 (16)	0.4091 (5)	0.0173 (10)	

### Atomic displacement parameters (Å^2^)

**Table:**

	*U*^11^	*U*^22^	*U*^33^	*U*^12^	*U*^13^	*U*^23^
Cu1	0.0279 (4)	0.0496 (4)	0.0144 (3)	−0.0021 (4)	0.0076 (3)	0.0018 (3)
Cu2	0.0125 (3)	0.0561 (5)	0.0334 (4)	0.0066 (4)	0.0042 (3)	0.0033 (4)
Cl1	0.0223 (7)	0.0274 (6)	0.0276 (8)	−0.0018 (5)	0.0016 (6)	0.0035 (5)
N1	0.012 (2)	0.032 (2)	0.012 (2)	0.0020 (18)	0.0023 (17)	0.0003 (18)
N2	0.013 (2)	0.036 (2)	0.014 (2)	0.0013 (19)	0.0042 (16)	−0.001 (2)
N3	0.017 (2)	0.038 (3)	0.011 (2)	0.0014 (19)	0.0038 (18)	−0.0001 (18)
N4	0.018 (2)	0.040 (3)	0.011 (2)	0.004 (2)	0.0035 (18)	0.001 (2)
N5	0.014 (2)	0.028 (2)	0.022 (2)	0.0037 (19)	0.0043 (19)	−0.0005 (19)
C1	0.016 (3)	0.028 (3)	0.025 (3)	0.006 (2)	0.001 (2)	0.006 (2)
C2	0.019 (3)	0.030 (3)	0.012 (3)	0.002 (2)	0.003 (2)	0.002 (2)
C3	0.017 (3)	0.034 (3)	0.018 (3)	0.002 (2)	0.005 (2)	−0.001 (2)
C4	0.021 (3)	0.028 (3)	0.012 (3)	0.004 (2)	0.002 (2)	−0.002 (2)
C5	0.012 (2)	0.023 (2)	0.017 (3)	0.002 (2)	0.002 (2)	−0.0028 (19)
C6	0.014 (2)	0.024 (2)	0.012 (2)	0.000 (2)	0.0001 (19)	−0.002 (2)

### Geometric parameters (Å, °)

**Table:**

Cu1—N3^i^	1.958 (5)	N4—C6	1.354 (7)
Cu1—N1	2.038 (5)	N5—C1	1.339 (8)
Cu1—Cl1	2.4422 (15)	N5—C3	1.349 (7)
Cu1—Cl1^ii^	2.5090 (16)	N5—Cu2^vi^	1.931 (4)
Cu2—N2^iii^	1.921 (5)	C1—C2	1.377 (8)
Cu2—N5^iv^	1.931 (4)	C1—H1A	0.9300
Cu2—Cl1	2.4923 (18)	C2—C5	1.389 (8)
Cl1—Cu1^iii^	2.5090 (16)	C2—H2A	0.9300
N1—C6	1.325 (7)	C3—C4	1.383 (8)
N1—N2	1.360 (7)	C3—H3A	0.9300
N2—N3	1.323 (7)	C4—C5	1.395 (8)
N2—Cu2^ii^	1.921 (5)	C4—H4A	0.9300
N3—N4	1.327 (7)	C5—C6	1.464 (7)
N3—Cu1^v^	1.958 (5)		
			
N3^i^—Cu1—N1	133.4 (2)	C1—N5—C3	116.8 (5)
N3^i^—Cu1—Cl1	116.27 (15)	C1—N5—Cu2^vi^	120.1 (4)
N1—Cu1—Cl1	97.70 (14)	C3—N5—Cu2^vi^	122.9 (4)
N3^i^—Cu1—Cl1^ii^	106.89 (15)	N5—C1—C2	123.1 (5)
N1—Cu1—Cl1^ii^	100.51 (13)	N5—C1—H1A	118.5
Cl1—Cu1—Cl1^ii^	94.90 (6)	C2—C1—H1A	118.5
N2^iii^—Cu2—N5^iv^	152.3 (2)	C1—C2—C5	119.8 (5)
N2^iii^—Cu2—Cl1	101.13 (17)	C1—C2—H2A	120.1
N5^iv^—Cu2—Cl1	106.30 (16)	C5—C2—H2A	120.1
Cu1—Cl1—Cu2	123.48 (7)	N5—C3—C4	124.0 (5)
Cu1—Cl1—Cu1^iii^	94.90 (6)	N5—C3—H3A	118.0
Cu2—Cl1—Cu1^iii^	78.17 (5)	C4—C3—H3A	118.0
C6—N1—N2	104.9 (5)	C3—C4—C5	118.2 (5)
C6—N1—Cu1	142.2 (4)	C3—C4—H4A	120.9
N2—N1—Cu1	111.9 (4)	C5—C4—H4A	120.9
N3—N2—N1	108.7 (5)	C2—C5—C4	117.9 (5)
N3—N2—Cu2^ii^	128.9 (4)	C2—C5—C6	118.6 (5)
N1—N2—Cu2^ii^	122.1 (4)	C4—C5—C6	123.4 (5)
N2—N3—N4	110.1 (4)	N1—C6—N4	111.5 (5)
N2—N3—Cu1^v^	129.6 (4)	N1—C6—C5	128.8 (5)
N4—N3—Cu1^v^	120.3 (4)	N4—C6—C5	119.6 (5)
N3—N4—C6	104.8 (4)		

Symmetry codes: (i) *x*, −*y*+2, *z*−1/2; (ii) *x*, *y*+1, *z*; (iii) *x*, *y*−1, *z*; (iv) *x*−1/2, *y*−1/2, *z*; (v) *x*, −*y*+2, *z*+1/2; (vi) *x*+1/2, *y*+1/2, *z*.
